# BioGPS: building your own mash-up of gene annotations and expression profiles

**DOI:** 10.1093/nar/gkv1104

**Published:** 2015-11-17

**Authors:** Chunlei Wu, Xuefeng Jin, Ginger Tsueng, Cyrus Afrasiabi, Andrew I. Su

**Affiliations:** 1 Department of Molecular and Experimental Medicine, The Scripps Research Institute, La Jolla, CA 92037, USA; 2 Nanjing Xiexu MediaTechnology Co., Ltd., 180 Ruanjiandadao Rd #7-403, Nanjing, Jiangsu 210000, China

## Abstract

BioGPS (http://biogps.org) is a centralized gene-annotation portal that enables researchers to access distributed gene annotation resources. This article focuses on the updates to BioGPS since our last paper (2013 database issue). The unique features of BioGPS, compared to those of other gene portals, are its community extensibility and user customizability. Users contribute the gene-specific resources accessible from BioGPS (‘plugins’), which helps ensure that the resource collection is always up-to-date and that it will continue expanding over time (since the 2013 paper, 162 resources have been added, for a 34% increase in the number of resources available). BioGPS users can create their own collections of relevant plugins and save them as customized gene-report pages or ‘layouts’ (since the 2013 paper, 488 user-created layouts have been added, for a 22% increase in the number of layouts). In addition, we recently updated the most popular plugin, the ‘Gene expression/activity chart’, to include ∼6000 datasets (from ∼2000 datasets) and we enhanced user interactivity. We also added a new ‘gene list’ feature that allows users to save query results for future reference.

## INTRODUCTION

In the massive network of biomedical knowledge, genes sit in a central position. Consulting a gene portal to gain insights into specific genes has become an every-day routine for biomedical researchers. BioGPS is a unique gene portal because it emphasizes community extensibility and user customizability (1,2). Its community extensibility allows the broad base of users, instead of a limited number of developers, to expand the collection of gene resources to include newly created resources and make them available for other users. Its user customizability allows users, instead of developers, to pick relevant resources and define the layout of their own gene report pages. This is especially important because the landscape of available gene resources is growing exponentially and is becoming more specialized. For example, a structural biologist might be interested in viewing a 3D structure from PDB (3) and protein family information from PFAM (4); a geneticist might be interested in viewing a genome browser and dbSNP (5) and eQTL data associated with specific genes. Even the same researcher might be interested in different sets of resources for different projects. BioGPS allows users to customize their own gene portals in a unified interface and extend them if a new resource they need has not been added yet.

In addition, BioGPS allows us to build customized ‘sub-portals’ for our collaborators. For example, we built a customized portal at http://biogps.org/exrna for the extracellular RNA (exRNA) community (6). It has the same interface and functionality as the main BioGPS portal, but users can search genes within the exRNA scope, display a set of exRNA related resources, and explore exRNA-specific expression profiles. Additional sub-portals have been created focusing on circadian rhythm studies (7) and primary cells gene expression (8).

Since its initial release in 2008 (1), BioGPS has continued to gain popularity among the research community. On average, BioGPS receives over 132 000 page views each month from ∼14 500 unique users (usage monitored by Google Analytics, from 07/2012 to 08/2015). Over 9000 users have signed up for BioGPS user accounts (up from 5000 in 2013 paper). Because most functionality is available to users without accounts, over 70% of our overall usage comes from anonymous users. These visits come from a combination of new visitors (40%) and returning visitors (60%).

**Figure 1.**
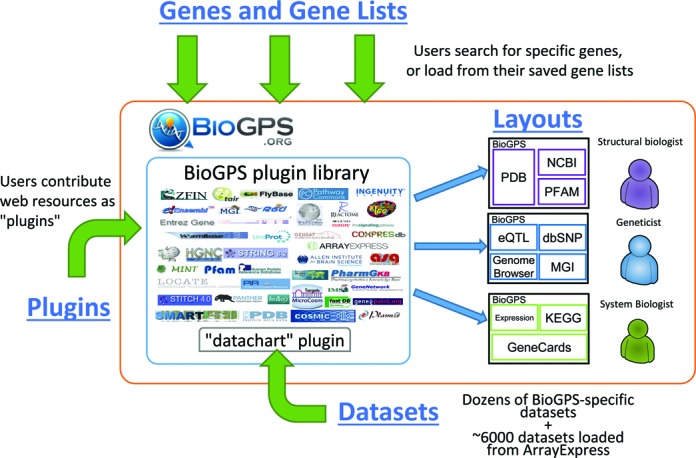
BioGPS is built upon four key components: plugins, layouts, genes/gene lists, and datasets. Plugins are the collection of gene-specific web-resources contributed by users. Layouts are the user-customized gene report pages with selected plugins from the plugin library. Genes are the entry point for users to search for genes and link to their relevant gene-report layouts; and gene lists are users’ saved genes from their previous queries. Datasets are those BioGPS-specific reference gene expression datasets and additional ∼6000 datasets loaded from ArrayExpress. They can be visualized in one of the most popular BioGPS plugins, ‘Gene expression/activity chart’ plugin (or ‘data chart’ plugin). These components enable BioGPS users to view their own customized gene report layouts with relevant resources or datasets, depending on their own research fields, e.g. structural biology, genetics or system biology.

The key features of BioGPS can be organized into four components: plugins, layouts, genes, and datasets (Figure 1). In this paper, we provide a progress report on these four key components, focusing on new features, such as ‘gene lists’ and the updated ‘dataset’ plugin.

## GENE ANNOTATION PLUGIN

The core functionality of BioGPS is built around ‘plugins’. Each plugin is a user-registered, gene-specific annotation web resource that is hosted externally. Any external web resource can be registered as a BioGPS plugin as long as the resource provides a direct URL to its gene-specific page using a common gene identifier, such as gene symbols, NCBI or Ensembl or MGI gene IDs, RefSeq IDs, Uniprot IDs, and Affymetrix probesets. BioGPS uses MyGene.info web services (2) to link these resources URLs to their associated genes. For example, the plugin for the Mouse Genome Informatics (MGI) (9) resource uses the following URL pattern: http://www.informatics.jax.org/marker/{{MGI}}. When this plugin is rendered for a user's gene (e.g. mouse Cdk2 gene), BioGPS automatically resolves the true URL as http://www.informatics.jax.org/marker/MGI:104772.

Plugins can be set as public (this is the default) or private (visible only to the owner). The BioGPS plugin library (http://biogps.org/plugin/) is the central hub in which users can browse all available plugins, either public or their own private plugins, or search for specific plugins.

Since our 2013 paper (2), the number of BioGPS plugins has increased from 480 to 642 (a 34% increase). Of all these plugins, the number that are publically-accessible has increased from 280 to 377 (a 35% increase). Of our 9097 registered users, 163 contributed one or more plugins (a 48% increase from previous 110 contributors). Supplementary Table S1 lists the new plugins added since our 2013 paper.

## GENE REPORT LAYOUT

The BioGPS plugin library contains all the gene-centric resources available to users; however, not all of these resources are relevant to every user at every moment. When users want to view resources for their favorite genes, they can choose which resources they want to view and how these resources should be displayed. Essentially, BioGPS allows users to customize their own ‘gene-report’ pages. Each customized gene-report page is called a ‘gene-report layout’ in BioGPS (or ‘layout’). Users can create multiple layouts for different scenarios, such as one for each project, or they can group commonly-used resources by categories like ‘pathways’, ‘literatures’ or ‘antibodies’. To help users get started, we created 13 public layouts that everyone can view; these layouts covering many popular resources of interest to researchers in general. The default gene-report layout contains the popular ‘Gene expression/activity chart’, ‘Gene identifiers’, and ‘Gene wiki’ plugins (Supplementary Figure S2), which provide gene-specific expression profiles and detailed gene summaries.

Since our 2013 paper (2), BioGPS users have created an additional 488 new layouts (a 22% increase from the previous 2200 layouts). Although the 13 default layouts are still the most popular, we have also observed that ∼1.7% of all layout requests are for user-created layouts. (Seventy percent of our overall usage comes from anonymous users who only have access to the default layouts.)

## GENE AND GENE LIST

BioGPS is a gene-centric portal. Our users typically begin by searching for specific genes. Under the hood, BioGPS utilizes the high-performance MyGene.info web services to match user input with the correct genes. MyGene.info provides up-to-date gene annotations and a rich query syntax to perform gene-based queries (2). BioGPS users can query for genes from nine common model organisms (*H. sapiens*, *M. musculus*, *R. norvegicus*, *D. melanogaster*, *C. elegans*, *D. rerio*, *A. thaliana*, *X. tropicalis* and *S. scrofa*) using over 50 supported identifiers or text fields, (e.g. Gene Ontology terms, KEGG pathway names or gene summaries), and genomic regions (e.g. searching for genes within the ‘chr1:151,073,054-151,383,976’ human genome interval). Currently, users performed over 50 000 gene searches each month on average. The popular searches are based on specific gene symbols, or NCBI/Ensembl/MGI gene IDs, as well as broad text-based searches (e.g. ‘kinase’ or ‘transcriptional factor’).

Since our last paper (2), we have added a new feature that allows users to save their search results as a ‘gene list’ in their profiles. Thus, users can load their saved gene lists directly into BioGPS and begin navigating without needing to remember their query and perform another search. Basic operations such as editing, intersections, and unions are also implemented for users’ convenience. Since its release, this feature has been proven to be popular among our users. Currently, over 6000 gene lists have been saved by over 1800 users (∼20% of all registered users). Given its popularity, we plan to add more features to the gene list component in the future, e.g. gene enrichment analysis and allowing plugins to take a gene list as the input.

## DATASET

Of all the registered plugins, the ‘Gene expression/activity chart’ plugin (or ‘data chart’ plugin; http://biogps.org/plugin/9/) is the most popular. This plugin is also one of a few plugins developed and maintained by the BioGPS team. The ‘data chart’ plugin is included in over 50% of all user-created layouts (1408 out of 2688). Given its popularity, we performed a major overhaul of this component recently to provide broader coverage of the datasets and enhanced user interactivity.

The initial release of the ‘data chart’ plugin (1) came with dozens of gene-expression reference datasets (10). We then expanded the dataset collection by pre-loading ∼2000 datasets from NCBI's GEO repository (11). We expanded the collection further by including ∼6000 datasets from EBI's ArrayExpress repository (12). These datasets come from nine common microarray platforms from humans, mice and rats (Supplementary Table S3). To help users navigate through these datasets, a dataset library interface has been added (http://biogps.org/dataset/). Similar to the plugin library we previously implemented, the dataset library allows users to list datasets by popularity, the date added, or specific tags (e.g. ‘breast cancer’). Users can further narrow down relevant datasets by searching for specific terms. From the list of datasets, users can then view details of the datasets or click the ‘view dataset’ button to explore the expression profiles from this dataset in the ‘data chart’ plugin.

In addition to the expression profile data, ArrayExpress provides manually curated sample annotations as part of the dataset metadata. We loaded these metadata into BioGPS so users can customize the visualization of expression profiles based on the sample annotations. For example, an expression profile can be rendered by treatment group versus control group instead of the default view, which only includes sample labels. Users can also choose to collapse samples within the same group (with the error-bar displayed) or simply group samples together for each group (Supplementary Figure S4). Currently, 74% of the loaded datasets (4395 out of 5914) contain manually curated sample annotations. We will continue synchronizing these metadata with ArrayExpress. Additional datasets will also be uploaded as our users create such requests through the issue tracker of our code repository.

## IMPLEMENTATION

The backend of BioGPS was built using the Django web framework with PostgreSQL as the database, and Elasticsearch as the full-text query engine. Its frontend was implemented with Sencha Ext JS and jQuery Javascript libraries. Up-to-date gene annotation data used for identifier-translation and plugin-rendering were accessed remotely via high-performance MyGene.info web services. The entire BioGPS application is hosted in Amazon's Elastic Compute Cloud (EC2).
